# Astragaloside IV Attenuates Experimental Autoimmune Encephalomyelitis of Mice by Counteracting Oxidative Stress at Multiple Levels

**DOI:** 10.1371/journal.pone.0076495

**Published:** 2013-10-04

**Authors:** Yixin He, Min Du, Yan Gao, Hongshuai Liu, Hongwei Wang, Xiaojun Wu, Zhengtao Wang

**Affiliations:** 1 Shanghai Key Laboratory of Complex Prescription, Institute of Chinese Materia Medica, Shanghai University of Traditional Chinese Medicine, Shanghai, P.R. China; 2 Department of Pharmacognosy, China Pharmaceutical University, Nanjing, P.R. China; 3 Unit of Immune Signaling and Regulation, Institut Pasteur of Shanghai, Chinese Academy of Sciences, Shanghai, P.R. China; University of Muenster, Germany

## Abstract

Multiple sclerosis (MS) is a chronic autoimmune neuroinflammatory disease found mostly in young adults in the western world. Oxidative stress induced neuronal apoptosis plays an important role in the pathogenesis of MS. In current study, astragaloside IV (ASI), a natural saponin molecule isolated from *Astragalus membranceus*, given at 20 mg/kg daily attenuated the severity of experimental autoimmune encephalomyelitis (EAE) in mice significantly. Further studies disclosed that ASI treatment inhibited the increase of ROS and pro-inflammatory cytokine levels, down-regulation of SOD and GSH-Px activities, and elevation of iNOS, p53 and phosphorylated tau in central nervous system (CNS) as well as the leakage of BBB of EAE mice. Meanwhile, the decreased ratio of Bcl-2/Bax was reversed by ASI. Moreover, ASI regulated T-cell differentiation and infiltration into CNS. In neuroblast SH-SY5Y cells, ASI dose-dependently reduced cellular ROS level and phosphorylation of tau in response to hydrogen peroxide challenge by modulation of Bcl-2/Bax ratio. ASI also inhibited activation of microglia both *in vivo* and *in vitro*. iNOS up-regulation induced by IFNγ stimulation was abolished by ASI dose-dependently in BV-2 cells. In summary, ASI prevented the severity of EAE progression possibly by counterbalancing oxidative stress and its effects via reduction of cellular ROS level, enhancement of antioxidant defense system, increase of anti-apoptotic and anti-inflammatory pathways, as well as modulation of T-cell differentiation and infiltration into CNS. The study suggested ASI may be effective for clinical therapy/prevention of MS.

## Introduction

Multiple sclerosis (MS) is a chronic autoimmune neuroinflammatory disease found mostly in young adults in the western world [1]–[2]. Focal lesions of the white matter with inflammation, demyelination, infiltration of immune cells, oligodendroglial death and consequent axonal damage are the major neurophysiological deficits in patients with MS [3]. Although many approved first-line drugs such as IFNβ, glatiramer acetate, mitoxantrone, and natalizumab, along with a number of treatments under investigation may curtail attacks or improve the progression of the disease [4], significant adverse effects including depression, infection, cardiotoxicity, nausea, and anemia have been found associated with the long-term therapy [5]. Moreover, as most of the available first-line drugs are either immunoregulators or immunosuppressants, so far there is no known cure for effectively halting neurodegeneration, and promoting remyelination and neuronal repair of the disease, which determine the final recovery of damaged neural system. Therefore, development of novel treatments with less adverse effects targeting not only immune system but also neuroregeneration or neuronal repair perhaps will benefit the therapy of the disease.

Although different mechanisms may result in the demyelination and neurodegeneration in MS, growing evidence indicates that oxidative stress plays the greatest role in the pathogenesis by contributing to myelin and oligodendroglia degeneration that finally leading to neuronal apoptosis [6]. Remarkably elevated oxidants have been found [7]–[9] in serum or cerebrospinal fluid (CSF) of MS patients as well as that in rodents induced with experimental autoimmune encephalomyelitis (EAE), the animal model of MS [10]–[11].The generation of free radicals, mostly by infiltrated monocytes and activated residential microglial cells, leads to a disruption of neuronal membrane integrity by interacting with the lipids, proteins, and nucleic acids and thus results in neuronal damage [12]–[14]. Additionally, weakened cellular antioxidant defense systems in the central nervous system (CNS) and enhanced vulnerability to oxidative stress effects in MS may increase damage [15]. Therefore, treatment with antioxidants might theoretically prevent against the damages and improve the survival of neuronal tissues in MS.

Astragaloside IV (ASI) is a small molecular saponin found in *Astragalus membranaceus* (Fisch.) Bge, which is a widely used herb in China. The herb shows anti-oxidation effects by inhibition of free radicals, reduction of lipid peroxidation and elevation of antioxidants enzymes [16]. Diverse pharmacological activities have been found to be exerted by the molecule such as anti-inflammation [17], anti-infarction [18], anti-hypertension [19], anti-diabetes [20], myocardial protection and anti-heart failure [21]. In addition, the natural compound has shown anti-oxidative effect in various cells, including human umbilical endothelial cells [22], rat adrenal pheochromocytoma PC12 cells [23], and rat H9C2 myocardiac cells [24]. Moreover, neuroprotective effect of ASI has been found by promoting axonal regeneration and the reconstruction of neuronal synapses [25]. Since oxidative stress is one of the major factors accounts for the pathogenesis of MS, we speculated that ASI, the neuroprotective antioxidant, might contribute to the prevention of MS progression. To testify the hypothesis, in present study C57BL/6 mice induced with EAE were treated with ASI. The results showed that ASI prevented the aggravation of EAE by counteracting oxidative stress and its effects at multiple levels, which suggested ASI may be effective for clinical therapy/prevention of MS.

## Materials and Methods

### Ethics Statements

All the animal experiments were carried out according to the protocol approved by Animal Care and Use Committee of Shanghai University of Traditional Chinese Medicine (Protocol # 11051). Subcutaneous injection and intracardial (i.c.) perfusion of mice were conducted after anesthetizing the animals with isoflurane and urethane, respectively, and all efforts were made to minimize suffering.

### EAE Induction and ASI Treatment

EAE induction was conducted in 6-week-old female C57BL/6 mice as described previously [26]. In brief, each mouse received subcutaneous injection of 100 µl of complete Freund’s adjuvant containing 300 µg of MOG_35–55_ and 400 µg of heat-inactivated *Mycobacterium tuberculosis* H37RA. Pertussis toxin (200 ng/mouse) was given intraperitoneally (i.p.) on the day of immunization and again two days later. Clinical behavior of mice was scored daily according to the criteria used by Madusha Peiris et al [27].

Astragaloside IV treatment (20 mg/kg) was given i.p. daily from the day before MOG_35–55_ immunization and continued for 2 weeks. Meanwhile, methylprednisolone (MPD) served as positive control drug was administered i.p. at 20 mg/kg dosage consecutively from day 8 to day 10 after MOG_35–55_ immunization.

### Cell Culture and Treatments

Neuroblast cell line SH-SY5Y obtained from ATCC was maintained in DMEM/F-12 (1∶1 v/v) medium supplemented with 10% fetal bovine serum (FBS), 100 U/ml penicillin and 100µg/ml streptomycin at 37°C with 5% CO_2_. To examine the anti-oxidative effect of ASI, hydrogen peroxide (H_2_O_2_) injured cell model was used. Briefly, cells were seeded at 1×10^6^/well density into a 6-well plate and pre-treated with 10, 20 and 50 µM ASI for 1 hr. Thereafter, H_2_O_2_ was added to the medium to the final concentration of 100 µM. After co-treated for 24 hrs, cells were harvested and lysed for further western-blotting analysis.

Microglia cell line BV-2 purchased from ATCC was cultured in DMEM medium with 10% FBS, 100 U/ml penicillin and 100 µg/ml streptomycin at 37°C with 5% CO_2_. After seeded at a density of 1×10^6^/well into a 6-well plate and cultured overnight, cells were pre-treated with 10, 20 and 50 µM ASI for 1 hr followed by stimulation of IFNγ (100 ng/ml) for 24 hrs. Then cells were harvested and lysed for further analysis.

CD4+ T cells were isolated from spleens of EAE (21 days post-immunization) and control mice with magnetic beads according to methods described by the manufacturer (Meltenyi Biotec, Germany). Cells were seeded at a density of 1×10^5^/well in 200 µl RPMI-1640 medium in a 96-well plate supplemented with anti-CD3 (5 µg/ml), anti-CD28 (1 µg/ml), IL2 (20 ng/ml), mercaptoethanol (50 µM) and 5% FBS. After treated with 50 µM ASI for three days, the culture medium was collected and subjected to further ELISA analysis.

### Histopathology and Immunohistochemistry (IHC)

Animals were anesthetized with excessive 20% urethane and perfused intracardially with PBS followed by 4% paraformaldehyde. Coronal sections of brains or spinal cords at 20 µm thickness were obtained on a Leica 1950 cryostat. Double staining of Luxol fast blue and cresyl echt violet was used to assess demyelination of CNS. Briefly, sections were stained with Luxol fast blue, washed with 95% ethanol, and then placed in lithium carbonate. Nuclei of the neuronal cells were visualized with cresyl echt violet staining. IHC was performed as described previously [26]. Primary antibodies including anti-GFAP and anti-IbaI were incubated with sections at 4°C overnight. After thoroughly washed by PBS, the sections were further incubated with Alexa 488 or 594 conjugated secondary antibodies. Fluorescent images were taken by using an inverted fluorescent microscope (Olympus IX 81).

### Western Blot

Brain cortices of mice were homogenized in CellLytic™ MT mammalian tissue lysis reagent (Sigma, C3228) supplemented with protease inhibitor cocktail (Sigma, P3840) and phosphatase inhibitor cocktail 2 (Sigma, P5726). Afterwards, the homogenate was centrifuged at 12,000 rpm and 4°C for 10 min. Protein concentration of the supernatants was quantified by BCA method. Thirty microgram of each samples were loaded to a 12% SDS-PAGE gel. After electrophoresis, proteins were transferred to FluoroTrans® W PVDF membranes (Pall, 20685) by an electrophoretic transfer system (Bio-Rad). The membranes were blocked with 5% skim milk in PBST for 1 hr and then incubated with respective primary antibodies at 4°C overnight. After thoroughly washed by PBST, the membranes were further incubated with horseradish perioxidase conjugated secondary antibodies and visualized with enhanced chemiluminescence and WB detection reagents (Amersham Biosciences, Pitscataway, NJ).

### ROS Measurements

*In vivo* ROS level was assessed by hydroethidine (HE), which is oxidized into dihydroethidine (DHE) by superoxide. The fluorescence of DHE can be measured by a fluorescent reader. Briefly, mice were injected i.p. with 200 µl of 1 mg/ml HE 15 min prior to sacrifice. Then brain cortices of mice were dissected and homogenized at 1∶5 ratio (w/v) in CellLytic™ MT mammalian tissue lysis reagent supplemented with protease inhibitor cocktail and phosphatase inhibitor cocktail 2. The homogenate was centrifuged at 12,000 rpm and 4°C for 10 min. The fluorescent intensity of resultant supernatants was measured using a Varioskan flash spectral scanning multimode reader (Thermo, excitation 540 nm; emission 595 nm) and normalized to tissue weight.

Intracellular ROS in SH-SY5Y cells was detected by 2′,7′-dichlorodihydrofluorescein (DCFH, Sigma, D6883), which is oxidized into fluorescent dichlorofluorescein (DCF) by ROS. Cells were incubated with 10 µM DCFH for 30 min. The dye was then removed and replaced with Hanks’ Balanced Salt Solution (HBSS) with Ca^2+^ & Mg^2+^. Fluorescence of the cells was observed under inverted fluorescent microscope or measured immediately using a Varioskan flash spectral scanning multimode reader (excitation 485 nm; emission 535 nm).

### Evan’s Blue Dye Extravasation

Permeability of brain was examined by Evan’s blue (EB) dye extravasation method. Mice were i.p. injected with 400 µl of 0.8% EB in PBS 2 hr before i.c. perfusion with PBS. Hemisphere of the brains was weighed and homogenized in 1 ml of 50% TCA. After centrifugation at 12,000 rpm for 10 min, the resultant supernatants were collected and the fluorescence was measured at an excitation wavelength of 620 nm and an emission wavelength of 680 nm.

### Cytokine Quantification

The concentrations of IFNγ, TNFα, IL6, IL4, and IL17A in brain homogenates or cell culture medium were measured by ELISA kits (eBiosciences, San Diego, CA). Cytokine concentrations in respective samples were determined by standard curves prepared by recombinant cytokines of known concentrations.

### Biochemical Analysis

Brain cortices of mice were homogenized in PBS (1∶10, w/v) on ice. After centrifuged at 4000 rpm and 4°C for 15 min, supernatants of the homogenates were immediately subjected to kits to analyze the concentration of malondialdehyde (MDA) and activities of total super oxide dismutase (T-SOD) and glutathione peroxidase (GSH-Px) in accordance to manuals of manufacturer (Jiancheng Bioengineering Institute, Nanjing, China).

### Quantitative PCR

Total RNA of the hippocampus and spleen was extracted using RNazol according to the manufacturer’s manual (Takara, Dalian, China). After digestion with DNase I to eliminate trace amounts of DNA contamination, total RNA was reverse transcripted into cDNA with kit. Quantitative PCR was conducted by use of Taqman SYBR kit. Concentrations of target genes in the samples were quantified by standard curves generated with template plasmids containing fragments of the respective target genes. Afterwards, they were normalized to that of glyceraldehydes-3-phosphate dehydrogenase (GAPDH) in the same sample. All primers used were listed in table 1.

**Table 1:** Primer sequences for quantitative PCR.

Gene name	Primer sequence
GAPDH	FP : ATGTGTCCGTCGTGGATCTGA
	RP : ATGCCTGCTTCACCACCTTCT
GFAP	FP : CGCTGGCAGCTGAACTGAA
	RP : GAAGCTCCGCCTGGTAGACAT
CD11b	FP : GGTCGGCAAGCAACTGATTT
	RP : CAACTTGCATTATGGCATCCA
Sod1	FP : ACCAGTGCAGGACCTCATTTTAA
	RP : AGGTCTCCAACATGCCTCTCTTC
GSH-Px	FP : CCTTGCCAACACCCAGTGA
	RP : GGCACACCGGAGACCAAAT
RORγt	FP: GGAAACCAGGCATCCTGAAC
	RP: GCACTGCAGAAACTGGGAATG
T-bet	FP: GAGGTGTCTGGGAAGCTGAGA
	RP: GCCGTCCTTGCTTAGTGATGA
Foxp3	FP: AGGAAAGACAGCAACCTTTTGG
	RP: GGCCACTTGCAGACTCCATT

### Statistical Analysis

Statistical analyses were carried out by GraphPad Prism 5 (GraphPad Software, La Jolla, CA). Repeated measures analysis of variance (ANOVA) was performed to determine the effects of treatments between groups in terms of EAE score. Pairwise comparisons among groups were performed by one-way ANOVA followed by Tukey’s posthoc test. All data in the graphs were presented as mean±standard error of the mean.

## Results

### ASI Reduced Severity of EAE Mice

**Figure 1:**
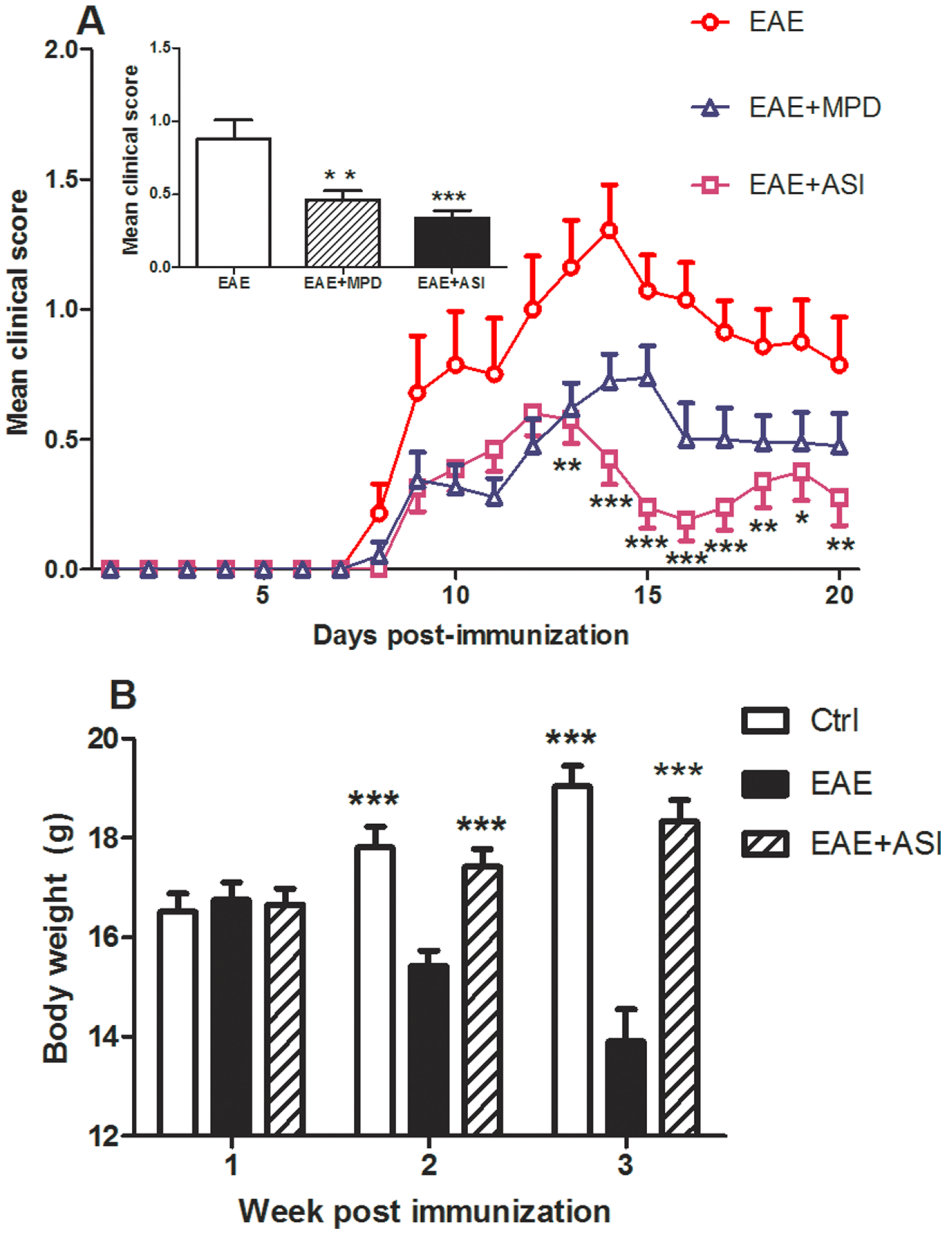
Astragaloside IV attenuated EAE progression. A, ASI treatment given at 20/kg/day i.p. from one day before MOG_35–55_ immunization and continued for 14 days significantly reduced mean clinical score of EAE mice. Inset denoted average clinical score daily of EAE mice received MPD (20 mg/kg/day, i.p. from day 8 to day 10 post-immunization), ASI or no treatment. B, ASI treatment prevented against body weight loss of EAE mice. MPD, methylprednisolone; ASI, astragaloside IV. All data are presented as mean±standard error of the mean (n = 17). *, p<0.05; **, p<0.01; ***, p<0.001.

Generally, onset of EAE disease was 8 days after MOG_35–55_ immunization and limb defect of mice attained its peak on day 14 post-immunization in mice without treatment (Fig. 1A). Afterwards, the behavioral symptom of mice showed slightly improvement in the following days. When MPD (20 mg/kg) was administered, the developmental episode in the mice was similar to that in EAE group without treatment as the onset of disease was still around 8 days. After induction, the limb defect of animals reached its top score on day 14 post-immunization. However, the extent of severity in MPD treated group mice was remarkably alleviated. ASI treatment at 20 mg/kg dosage from the day before immunization to day 14 post-immunization altered the disease developmental pattern. The compound not only prevented the deterioration of EAE from day 12, but also reduced the average behavioral score of mice. From day 13 post-immunization, behavioral score of ASI treated EAE mice was significantly lower compared with the control group mice. Moreover, although shown a slight relapse after cease of treatment, the behavioral symptom in ASI treated mice did not deteriorate too much. And the disease in treated mice remitted again from day 20 post-immunization. In addition, the average clinical score of ASI treated mice was even lower than that of MPD treated mice during the incidence of the disease. Compared with EAE mice without treatment, both MPD- and ASI-treated mice showed significantly decreased clinical score during the incidence of disease (p<0.01 and p<0.001, respectively, shown in inset of Fig. 1A). To confirm the results of clinical score, we monitored the body weight of mice weekly. As illustrated in Fig. 1B, mice showed persistent loss of body weight with the progression of EAE if received no treatment. However, ASI treatment prevented against body weight loss of EAE mice, which indicated the preventive effect of the compound.

### ASI Attenuated Demyelination and Neuroinflammation of EAE Mice

**Figure 2:**
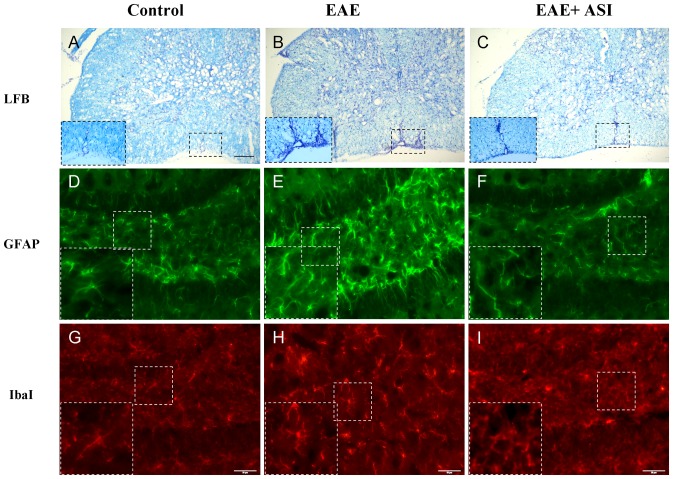
Astragaloside IV reduced demyelination and neuroinflammation. A, B, C, ASI lessened demyelination of EAE mice stained by LFB and prevented infiltration of monocytes into spinal cords of EAE mice. Insets denoted nuclei of monocytes stained by cresyl echt violet in anterior median fissure of spinal cords. D, E, F, ASI decreased activation of astrocytes labeled by GFAP in dentate gyrus of EAE mice. Insets indicated enlarged astrocytes in control, EAE and EAE+ASI mice. G, H, I, ASI inhibited activation of microglial cells labeled by Iba I in dentate gyrus of EAE mice. Insets indicated enlarged microglia in control, EAE and EAE+ASI mice. Error bars in LFB staining denoted 70 µm, while those in Iba I staining represented 50 µm. ASI, astragaloside IV.

To examine whether ASI treatment could lessen demyelination of CNS, LFB staining was conducted on spinal cord sections. As shown in Fig. 2A–C, compared with control mice, EAE mice displayed the severest demyelination. In contrast, those mice treated with ASI exhibited significantly improved symptom demonstrated by the enhanced LFB staining. Moreover, EAE induction led to much more infiltration of monocytes into spinal cord, especially in anterior median fissure region, which were stained by cresyl echt violet (insets in Fig. 2A–C). ASI administration inhibited the infiltration of monocytes to the same region.

To assess if ASI could attenuate neuroinflammtion indicated by activation of glial cells, we conducted fluorescent IHC by using GFAP and Iba I antibodies to label astrocytes and microglia, respectively (Fig. 2D–I). In dentate gyrus of hippocampus, significantly activated astrocytes as well as microglia were found in EAE mice. Those activated glial cells showed enlarged cell bodies, retracted processes and increased GFAP or Iba I immunoreactivity. However, in ASI treated mice, those cells looked like normal ones in resting condition. Both GFAP and Iba I immunoreactivities were reduced compared with that in EAE mice without any treatment. Although a few number of activated microglia still could be found in ASI treated mice, most of the microglial cells showed ramified shape that suggested less occurrence of neuroinflammation.

### ASI Decreased ROS Stress in EAE Mice

**Figure 3:**
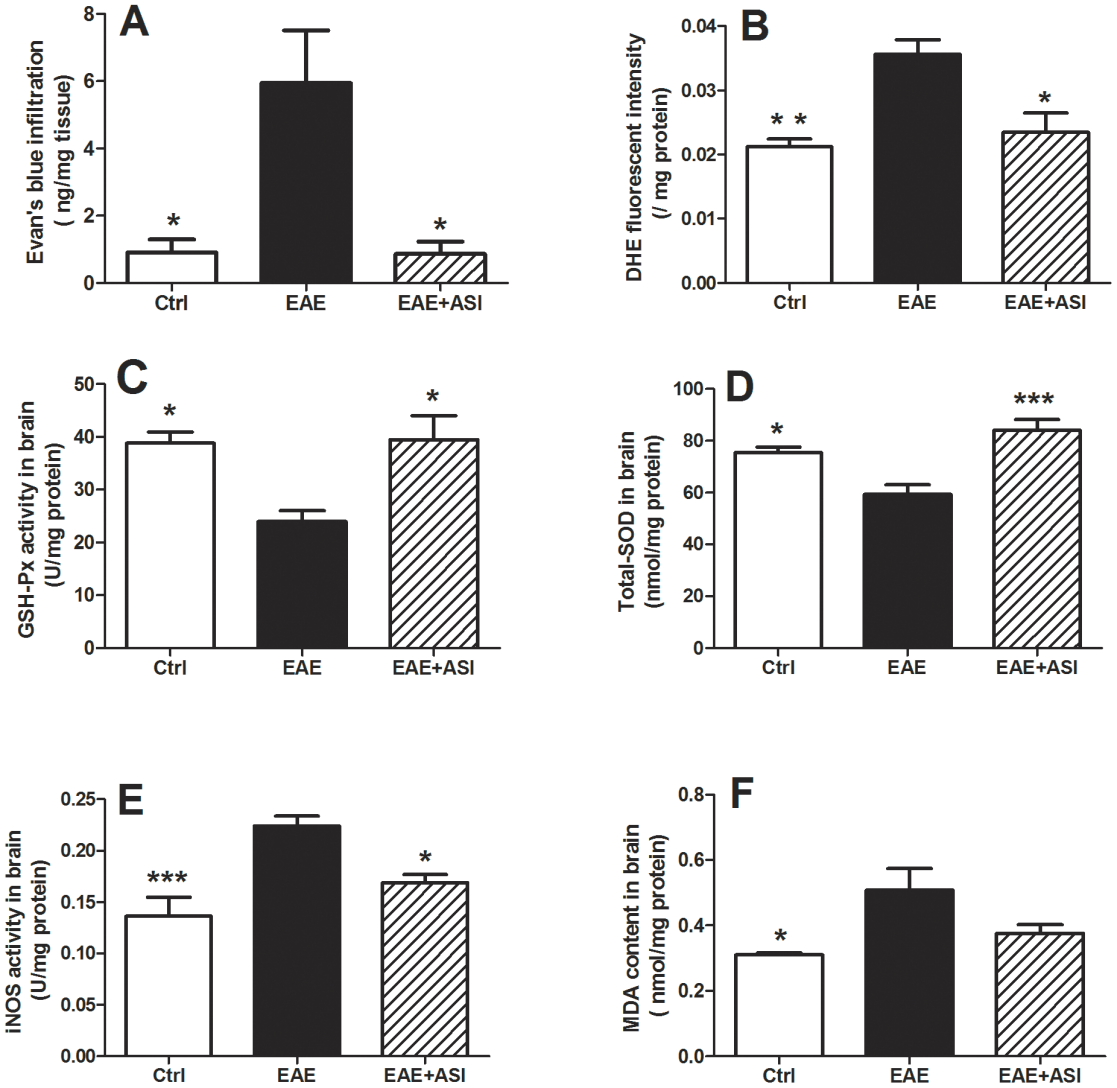
Astragaloside IV regulated BBB permeability and ROS levels. A, ASI prevented BBB leakage of EAE mice measured by Evan’s blue dye infiltration; B, ASI decreased in vivo ROS level of EAE mice analyzed by DHE fluorescent intensity; C, D, ASI resumed down-regulated anti-oxidative enzymes, GSH-Px and T-SOD in EAE mice; E, F, ASI prevented increase of iNOS and MDA in EAE mice. All data are presented as mean±standard error of the mean. n = 5 in each group. *, p<0.05; **, p<0.01; ***, p<0.001. ASI, astragaloside IV.

As opening of BBB and oxidative stress are known to be involved in the pathogenesis of EAE [28], we firstly analyzed the BBB permeability by measuring infiltrated Evan’s blue dye. In agreement with previous reports, EAE resulted in leakage of BBB even three weeks post-immunization (p<0.05, Fig. 3A). ASI treatment reversed the increased BBB leakage. Sequentially, we estimated *in vivo* ROS levels in CNS of mice by measuring infiltrated DHE. Compared with the control mice, ROS level in the cortices of EAE mice was much higher (p<0.01, Fig. 3B). As a result, more MDA, the breakdown product of oxidation of polyunsaturated fatty acids served as a reliable oxidant marker of oxidative stress-mediated lipid peroxidation [29], were generated in EAE mice (p<0.05, Fig. 3F) as well as iNOS (p<0.001, Fig. 3E). Opposite to increased ROS level, MDA and iNOS production, GSH-Px and total SOD activities in EAE mice were down-regulated (p<0.05, Fig. 3C and D). ASI administration reversed ROS and anti-oxidative enzymes levels in EAE mice as less ROS indicated by infiltrated DHE concentration (p<0.05, Fig. 3A) were found in cortices from ASI treated mice as well as less MDA (p = 0.08, Fig. 3B) and iNOS (p<0.05, Fig. 3E) were generated. Furthermore, GSH-Px and total SOD activities in ASI treated mice were significantly elevated compared to that in EAE mice (p<0.05 and p<0.001, respectively, Fig. 3C and D). Moreover, total SOD activity in ASI treated mice was increased even higher than that in control mice in spite of no statistical significance.

### ASI Affected mRNA Expressions of Hippocampal GFAP, CD11b but not Sod1 and Gpx1

**Figure 4:**
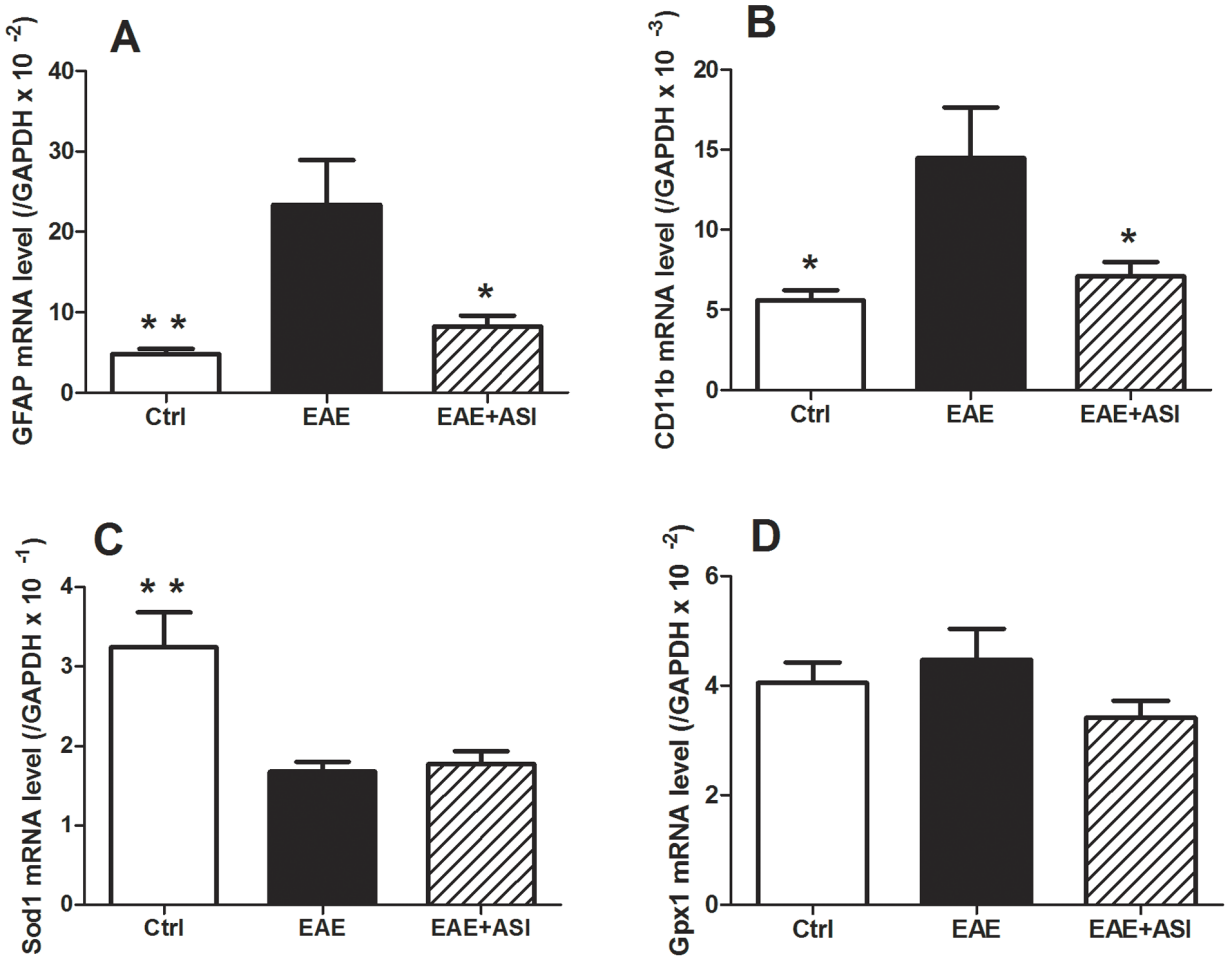
Effects of astragaloside IV on mRNA expressions of neuroinflammatory biomarkers and anti-oxidative enzymes. A, ASI decreased GFAP mRNA expression in cortices of EAE mice. B, ASI reduced CD11b mRNA expression in cortices of EAE mice. C, D, ASI did not change mRNA expressions of Sod1 and Gpx1 in cortices of EAE mice. All data are presented as mean±standard error of the mean. n = 5 in each group. *, p<0.05; **, p<0.01. ASI, astragaloside IV.

In consistent with IHC pattern, mRNA expression level of GFAP in hippocampi of EAE mice was significantly elevated (p<0.01, Fig. 4A) as well as that of CD11b, the microglial marker in CNS (p<0.05, Fig. 4B). When treated with ASI, mRNA expression levels of both GFAP and CD11b were down-regulated remarkably (p<0.05, Fig. 4A–B), which indicated the decline of neuroinflammation. Not surprisingly, mRNA expression of Sod1, one of the subtypes of SOD, was reduced in EAE mice (p<0.01, Fig. 4C). But glutathione peroxidase-1 (Gpx1) mRNA was not changed (Fig. 4D). To our surprise, ASI treatment had not altered the mRNA expression levels of both Sod1 and Gpx1.

### ASI Modulated mRNA Expressions of Splenic T-bet, RORγt, and Foxp3

**Figure 5:**
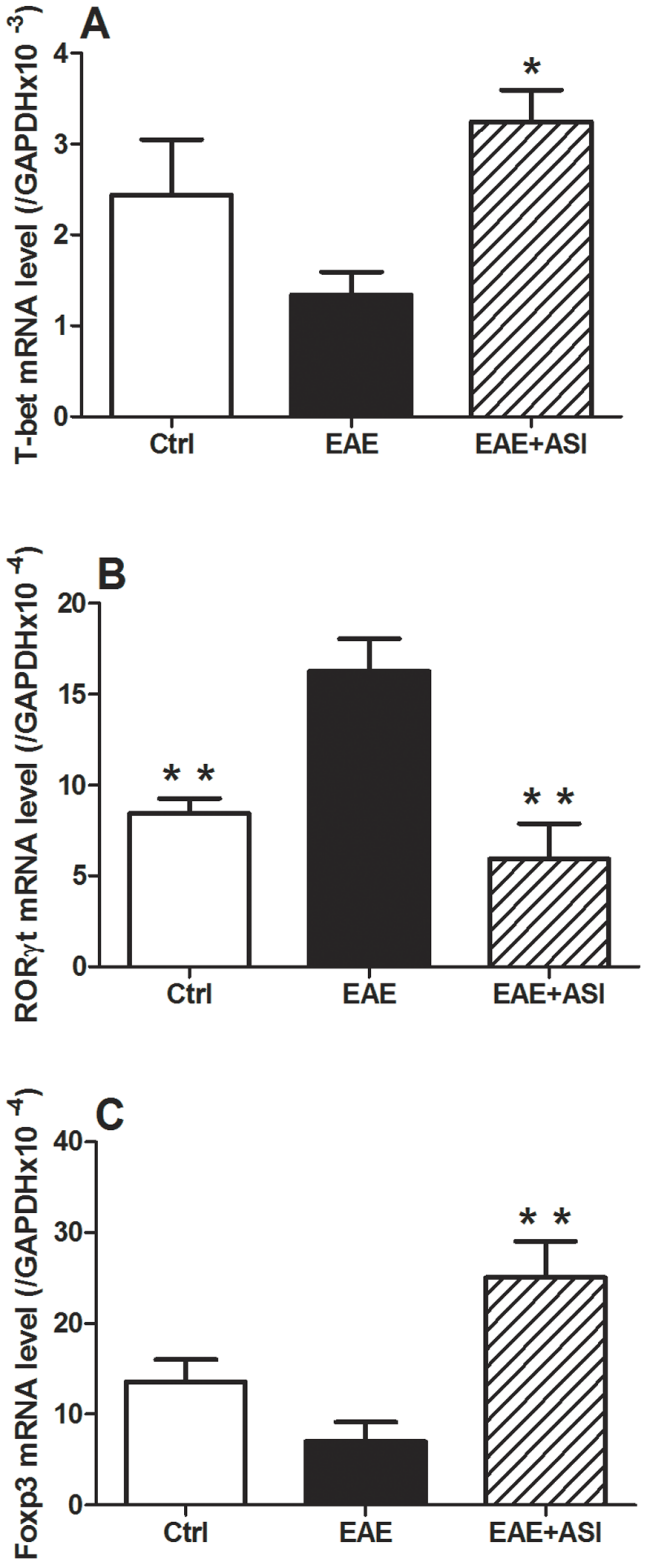
Effects of astragaloside IV on splenic RORγt, T-bet and Foxp3 mRNA expressions of EAE mice. All data are presented as mean±standard error of the mean. n = 5 in each group. *, p<0.05; **, p<0.01. ASI, astragaloside IV.

To investigate if ASI could regulate T cell differentiation, we examined splenic RORγt, T-bet and Foxp3 mRNA expressions using qPCR approach. As illustrated in Fig. 5, EAE caused significant elevation of RORγt gene expression (p<0.01). In contrast, T-bet and Foxp3 mRNA expressions were inhibited a little bit (Fig. 5 A and C). ASI administration decreased RORγt mRNA expression (p<0.01). Meanwhile, ASI enhanced significant increase of T-bet and Foxp3 mRNA levels (p<0.05 and p<0.01, respectively).

### ASI Regulated Cytokine Profile of EAE Mice

**Figure 6:**
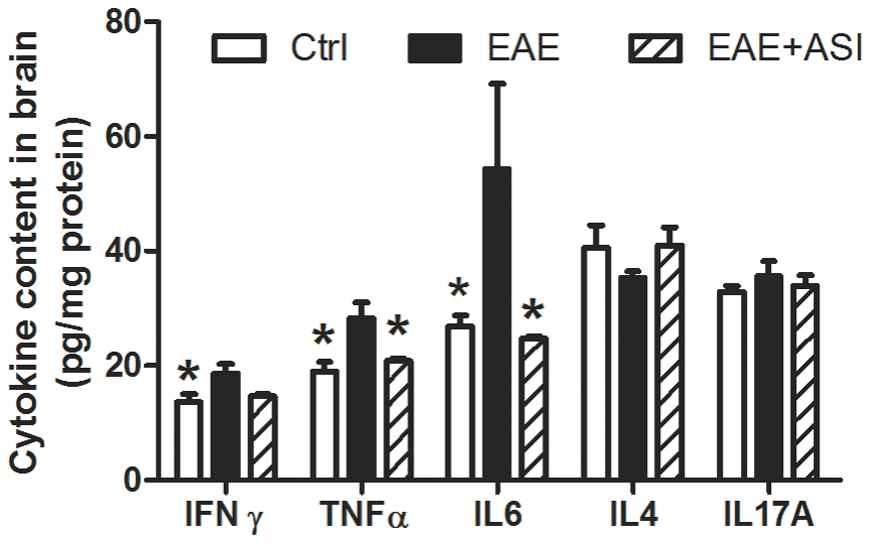
Effects of astragaloside IV on cytokine profile in CNS of EAE mice. All data are presented as mean±standard error of the mean and compared with EAE group. n = 5 in each group. *, p<0.05.

To assess the effect of ASI on the cytokine expression profile, which was mainly secreted by inflammatory cells, cortices of mice three weeks post-immunization were homogenized and subjected to ELISA assays. To our surprise, concentration of IL17A, the cytokine secreted by infiltrated Th17 cells, was not changed in EAE mice as our predicted (Fig. 6). Similarly, IL4, one of the Th2 cytokines, showed no remarkable alteration. But Th1 cytokines, IFNγand TNFα, were elevated (p<0.05). Meanwhile, IL6 was also up-regulated. After ASI treatment, increased expressions of IFNγ, TNFα and IL6 were all down-regulated significantly (p<0.05).

### ASI Modulated Expression of Apoptotic Proteins in CNS

**Figure 7:**
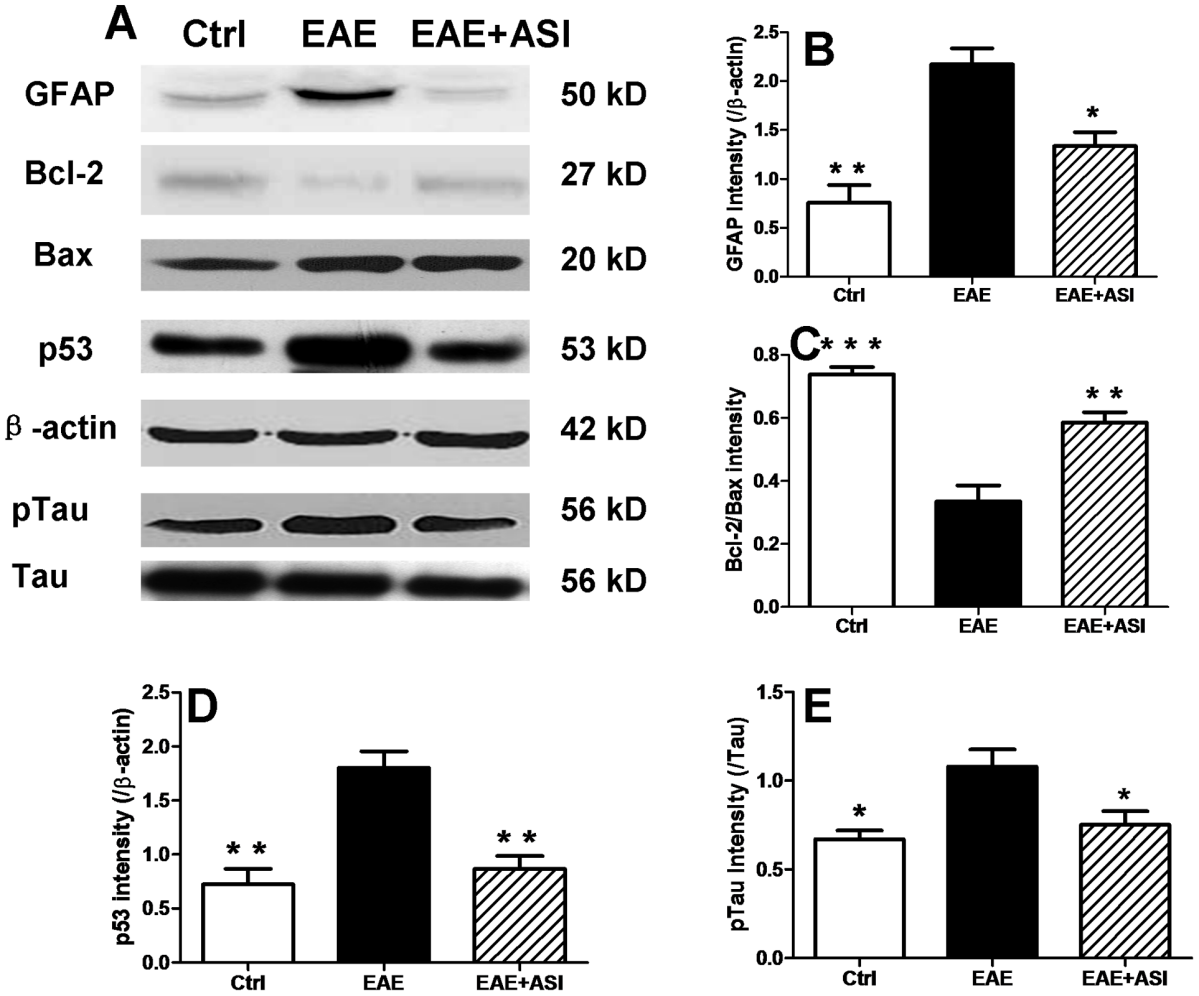
Effects of astragaloside IV on apoptotic proteins. A, western blots of GFAP, Bcl-2, Bax, p53, p-tau in cortices of EAE mice treated with astragaloside IV. B-E, gray intensity analysis of GFAP, Bcl-2, Bax, p53 and p-tau. All data are presented as mean±standard error of the mean and compared with EAE group. n = 5 for each group. *, p<0.05; **, p<0.01; ***, p<0.001.

To evaluate the impact of ASI administration on neuronal damage, expression levels of proteins associated with apoptosis in cortices of mice were analyzed (Fig. 7). Not surprisingly, GFAP, the astrocytic marker, was remarkably increased in EAE mice without any further treatment (p<0.01, Fig. 7A and B). p53, the pro-apoptotic protein, was also robustly up-regulated in EAE mice (p<0.01, Fig. 7A and D). As a result, the phosphorylated tau indicated neuronal damage was found to be elevated significantly in those mice (p<0.05, Fig. 7A and E). Bax, one of the members of Bcl-2 family, was slightly increased (Fig. 7A). However, on the contrary, Bcl-2 was decreased markedly and its ratio to Bax was down-regulated prominently (p<0.001, Fig. 7A and B). When treated with ASI, neuroinflammation indicated by up-regulated GFAP immunoreactivity was significantly reduced (p<0.05, Fig. 7A and B). Meanwhile, p53 in the CNS of ASI treated mice was decreased remarkably (p<0.01, Fig. 7A and D). Conversely, Bcl-2/Bax ratio in the cortices was elevated compared to that in EAE mice without any treatment (p<0.01, Fig. 7A and C). As a result, phosphorylation of tau in ASI treated mice was attenuated, which suggested less axonal damage (p<0.05, Fig. 7A and E).

### ASI Decreased Oxidative Stress in SH-SY5Y Cells and iNOS Expression in BV-2 Cells

**Figure 8:**
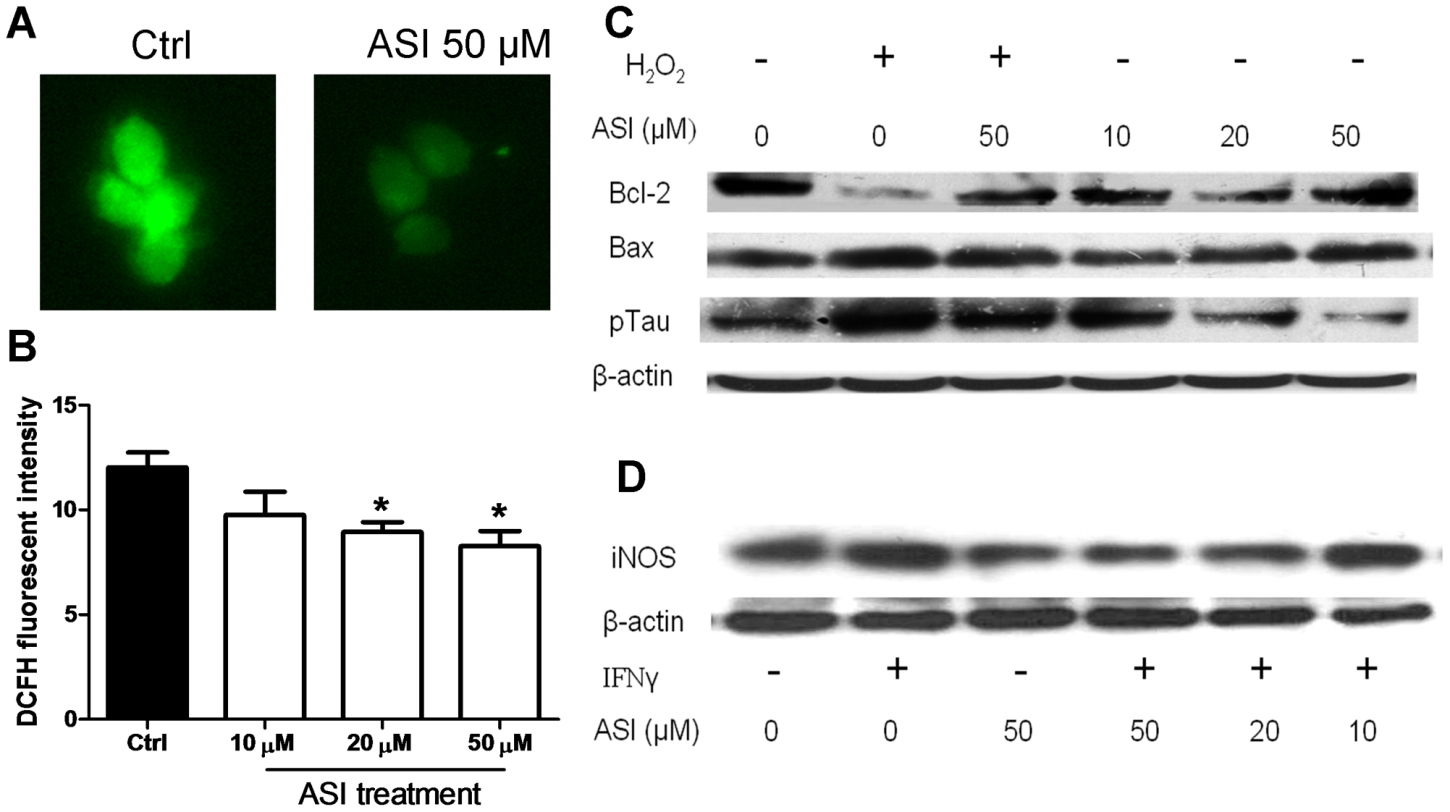
Astragaloside IV reduced oxidative stress in SH-SY5Y cells and decreased iNOS expression in BV-2 cells upon IFNγ stimulation. A, SH-SY5Y treated with ASI (50 µM) for 24 hr reduced cellular DCF fluorescence. B, SH-SY5Y treated with different doses of ASI for 24 hr inhibited cellular DCF fluorescent intensity (n = 5). C, SH-SH5Y cells were pretreated with ASI for 1 hr followed by challenge of H_2_O_2_ (100 µM) for 24 hr. D, BV-2 cells were pretreated with ASI for 1 hr followed by stimulation of IFNγ (100 ng/ml) for 24 hr. ASI, astragaloside IV.

To examine if ASI has some direct effects on oxidative stress of neuronal cells, SH-SY5Y cells were incubated with DCFH for 30 min after pre-treated with ASI overnight. As illustrated in Fig. 8A, DCF fluorescence in ASI (50 µM) treated cells were significantly lower than that in the cells without any treatment. Moreover, ASI dose-dependently reduced DCF fluorescent intensity in normal SH-SY5Y cells (Fig. 8B). When subjected to exogenous oxidative stress challenge, for instance, 100 µM H_2_O_2_, more phosphorylated tau was found in the cells as well as elevated Bax molecules (Fig. 8C). On the contrary, Bcl-2 was shown to be reduced. After co-treated with 50 µM ASI, down-regulation of Bcl-2 was abolished without simultaneous decrease of Bax. As a result, phosphorylation of tau induced by H_2_O_2_ was alleviated. Moreover, when there’s no exogenous oxidative stress, ASI dose-dependently (10–50 µM) induced Bcl-2 expression which resulted in less phosphorylation of tau. In BV-2 cells, when stimulated with 100 ng/ml of IFNγ, one of the Th1 cytokines, more iNOS proteins were induced as compared to the cells without any treatment (Fig. 8D). ASI treatment dose-dependently prevented against up-regulation of iNOS of BV-2 cells in response to IFNγ stimulation.

### ASI Inhibited Secretion of Th1 and Th17 Cytokines from CD4+ Cells

**Figure 9:**
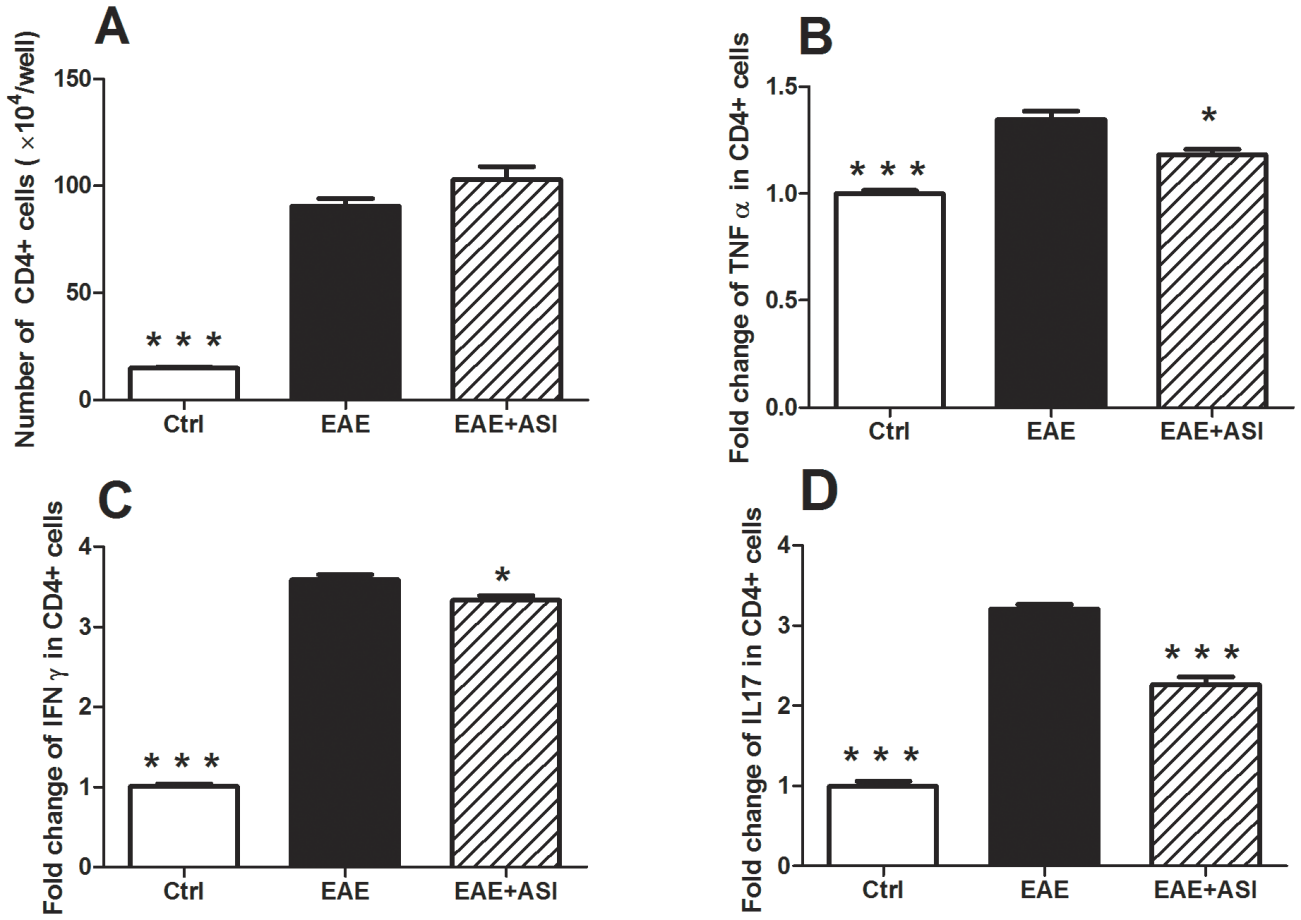
Effects of astragaloside IV on proliferation and cytokine secretion of CD4+ T cells. A, ASI did not prevent proliferation of CD4+ T cells isolated from EAE mice. B, ASI inhibited TNFα secretion from CD4+ T cells isolated from EAE mice. C, ASI reduced IFNγ production of CD4+ T cells isolated from EAE mice. D. ASI decreased IL17 secretion from CD4+ T cells isolated from EAE mice. Control CD4+ T cells were isolated from spleens of normal C57 BL/6 mice. EAE mice used for CD4+ T cell isolation were induced with MOG_35–55_ for 21 days. ASI, astragaloside IV. All data are presented as mean±standard error of the mean and compared with EAE group. n = 5 for each group. *, p<0.05; ***, p<0.001.

Since T helper cells, especially Th1 and Th17 cells, play vital role in the pathogenesis of EAE, we investigated cytokine profile changes of CD4+ cells after ASI treatment. As shown in Fig. 9A, CD4+ cells isolated from EAE mice (21 days post-immunization) proliferated much faster than that from control mice (p<0.001). Moreover, levels of pro-inflammatory cytokines secreted by EAE CD4+ cells including IFNγ, TNFα and IL17 were significantly higher compared with that of the control (p<0.001, Fig. 9B–D). ASI treatment (50 µM) did not prevent the proliferation of but suppressed IFNγ, TNFα and IL17 secretion from the CD4+ cells isolated from EAE mice (p<0.01 and p<0.001, respectively, Fig. 9B–D).

## Discussion

In current study, ASI administration significantly prevented against aggravation of neuropathology of EAE mice. Further studies disclosed that ASI intervened progression of the disease perhaps via anti-oxidative stress at multi-levels. In EAE mice treated with ASI, production of ROS in CNS was decreased remarkably concurrent with robustly elevated total SOD and GSH-Px activities. Meanwhile, neuroinflammation indicated by activated astrocytes and microglia was significantly alleviated as well as cytokines secreted by the inflammatory cells. As a result, axonal damage indicated by reduction of phosphorylated tau and MDA was attenuated markedly. Further studies conducted *in vitro* exposed that ASI could eliminate intracellular ROS and reduce damage to neuronal cells resulted from exogenous ROS perhaps via regulation of Bcl-2/Bax ratio, especially increase of Bcl-2 expression, and inhibition of iNOS expression in residential microglial cells.

The level of reactive oxygen species (ROS) is known to be enhanced in MS [30], which consequently causes increased permeability of the BBB [31]–[32]. Similarly, opening of the BBB and oxidative stress are known to be involved in the pathogenesis of EAE, the animal model of MS [28], [33]. In agreement with previous studies, we observed the leakage of BBB and elevated ROS in CNS of EAE mice (Fig. 3) as well as enhanced demyelination and neuroinflammation (Fig. 2), which confirmed the association between ROS caused opening of BBB and neural damage. When treated with ASI, leakage of BBB and elevation of ROS in EAE mice were suppressed remarkably and almost resumed to normal levels (Fig. 3). As aforementioned, ASI showed anti-oxidative effect in human umbilical endothelial cells [22], whether it has similar influence on brain microvessel endothelial cells, the main component of BBB, has not been disclosed yet. Further studies to investigate if ASI has such an effect on brain microvessel endothelial cells are undergoing in our lab, which may provide a direct evidence of the protective effect of ASI on the integrity of BBB.

T cell infiltration is one of the crucial features of EAE [34]. CD4+ T helper cells, including Th1 and Th17, and T regulatory (T-reg) Foxp3+ cells are key players for the EAE pathogenesis and recovery [35]. The differentiation of T cells depends on the transcription factors T-bet (Th1), RORγt (Th17) and Foxp3 (T-reg). The signature cytokines of these different subsets of T cells were modulated by the transcription factors. Th1 cytokines such as IFNγ and TNFα are regulated by T-bet [36], Th17 cytokines IL17, IL22 or IL21 by RORγt, while T-reg cytokines IL10 and TGFβ by Foxp3 [37]. Our findings indicated that ASI could regulate the splenic expression of all of the three mentioned transcription factors at mRNA levels, especially Foxp3 (Fig. 5). In agreement with our findings, ASI has been reported to antagonize the down-regulated expressions of Tregs cell phenotypes caused by high mobility group box 1 protein [38]. Consistent with the qPCR results, western blot result also showed increased CD3+ T cells infiltrated into spinal cord of EAE mice (Fig. S1, p<0.01). As been found to bind to the membranes of all mature T-cells and to be present at all stages of T-cell development, CD3 is a useful marker for T-cells in immunohistochemistry. In spinal cord of the mice treated with ASI, there was less CD3 expression (p<0.05). Moreover, in vitro experiments displayed that ASI treatment suppressed Th1 and Th17 cytokine secretion from CD4+ cells isolated from EAE mice (Fig. 9). Our unpublished data also showed that ASI inhibited Th1 and Th17 cytokine secretion by CD4+ T cell stimulated with MOG. Therefore, our findings suggested that ASI perhaps intervened the differentiation of T cells and thus prevented them from infiltrating into CNS.

As ROS was elevated in the progression of EAE, anti-oxidants such as SOD and GSH-Px were found synchronously to be gradually decreased, especially at early stages of the disease [39]. SOD catalyzes the dismutation of superoxide anion to hydrogen peroxide. For GSH-Px, it reduces lipid hydroperoxides to their corresponding alcohols and free hydrogen peroxide to water. The process is important for the relief of the oxidative damage to the organs, especially nervous system. Reduction of the enzymes causes a variety of toxic effects, including lipid peroxidation indicated by MDA. In consistence with the reports, we observed remarkably decreased SOD and GSH-Px activities in the brains of EAE mice accompanied with elevated MDA concentration. Meanwhile, the mRNA level of SOD1, the major isoenzyme of SOD, was up-regulated. After treatment with ASI, the down-regulated SOD and GSH-Px activities were recovered although mRNA levels of both enzymes were not enhanced synchronously, which suggests that ASI may modulate the activities of the enzymes at transcriptional or degradative levels.

The balance of pro- and anti-apoptotic proteins of Bcl-2 family plays an important role in the control of apoptotic cascade of cells. Thus, the ratio of Bcl-2 to Bax perhaps is a better determinant for cell survival than the absolute concentration of either protein alone [40]–[41]. The pro-apoptotic proteins of Bcl-2 family have been found to participate in neuronal death [42]. In current study, we found increased Bax in both *in vivo* and *in vitro* models (Fig. 6 and Fig. 7). When subjected to ASI treatment, the decrease of Bcl-2, the anti-apoptotic protein, was prevented although the level of it did not exceed that in normal condition. In contrast, ASI treatment did not reduce Bax expression in either EAE mice or SH-SY5Y cells challenged with H_2_O_2_. Therefore, the increased ratio of Bcl-2/Bax in ASI treated group may be due to the preventive effect of ASI on the degradation of Bcl-2 protein or the up-regulation of mRNA of Bcl-2. Our unpublished data showed that ASI could increase the phosphorylation of Bcl-2 at Ser 70 through MAPK pathway. As phosphorylation of Bcl-2 inhibited its ubiquitinization and thereby conferred resistance to cellular apoptosis [43], anti-apoptotic capacity of ASI on neurons perhaps mainly via MAPK mediated Bcl-2 phosphorylation pathway.

p53, the tumor suppressor molecule, is important in inducing DNA repair, cell cycle arrest or apoptosis after genotoxic stress [44]. Endogenous factors, such as NO and its metabolites, can induce p53-dependent neuronal cell apoptosis [45]–[46], in part by regulation of Bcl-2 and Bax [47]–[48]. In our work, we observed increased p53 as well as iNOS activity in EAE mice brain (Fig. 6). Meanwhile, Bcl-2 was decreased accompanied with elevated Bax. ASI treatment significantly reversed the process in brain cortices of EAE mice. Therefore, we speculated ASI might also regulate p53 via Bcl-2/Bax pathway, which is still lack of evidence currently.

Tau protein binds to and stabilizes microtubules in a phosphorylation-dependent pattern [49]. Study shows tau is engaged in modulation of anterograde axonal transport by influencing the attachment/detachment rate of molecular motors along microtubules [50]. Hyperphosphorylation of tau results in detachment and accumulated unbound protein initiates its aggregation into toxic paired helical filaments [51]. Abnormally increased phosphorylated tau has been found to be associated with neuronal and axonal loss in EAE and MS [51]–[52]. In our study, phosphorylation of tau in EAE mice was attenuated by ASI treatment (Fig. 7). In SH-SY5Y cells, ASI alleviated phosphorylation of tau induced by H_2_O_2_ even under basal conditions. Apparently, ASI could prevent further axonal damage probably by inhibiting phosphorylation of structural proteins. The ratio of Bcl-2/Bax has been shown to be associated with phosphorylation of tau [53]. As mentioned above, the ratio of Bcl-2/Bax was elevated both *in vitro* and *in vivo* after ASI treatment. Thus protective effect of ASI on neuronal cells, at least partially, was conduced via regulation of Bcl-2/Bax pathway.

Microglia regarded as innate immune cells in CNS exert similar functions as peripheral macrophages [54]. They eliminate invading pathogens, remove deleterious debris, accelerate tissue repair and facilitate tissue homeostasis, therefore, help host defense [55]. However, sustained and uncontrolled activation of microglia can produce excessive detrimental substances, most notably nitric oxide, free radicals and proinflammatory cytokines that result in neuronal destruction [56]. In current study, prominently activated microglial cells were found at both protein and mRNA levels in CNS of EAE mice (Fig. 2 and Fig. 3). Moreover, increased iNOS activity and elevated IFNγ, TNFα and IL6 levels were found in cortices of EAE mice (Fig. 3 and Fig. 6). When given ASI treatment, levels of iNOS activity and those cytokines were down-regulated. In BV-2 cells, ASI dose-dependently reduced IFNγ-induced iNOS increase. Therefore, ASI could suppress microglial activation and thus reduce nitric oxide and pro-inflammatory cytokines produced in CNS.

As most of first-line drugs used for MS therapies currently are either immunoregulators or immunosuppressants, which are associated with risk of infection and organ toxicity more or less, more strategies are in need to develop new therapies to halt neurodegeneration, boost remyelination and neuronal repair while not just target immune system [5]. Although in present study we mainly reported anti-oxidative property of ASI, neuroregenerative effects of this natural compound had been recognized for a long time in terms of promoting axonal regeneration and the reconstruction of neuronal synapses [25]. The natural compound could protect neural cells against MPP+ or 6-OHDA-induced dopaminergic neurotoxicity [57]–[58]. In hippocampal neurons ASI was able to decrease the frequencies of synchronized spontaneous Ca^2+^ oscillations and spontaneous excitatory postsynaptic currents [59]. In current study, we found ASI significantly reduced H_2_O_2_-induced phosphorylation of tau in SH-SY5Y cells suggested less loss of neurons. All these findings indicate ASI, the small saponin molecule with both neural regenerative and anti-oxidative properties, perhaps could be a hopeful drug candidate, at least an accessory medicine for MS therapy or prevention.

**Figure 10:**
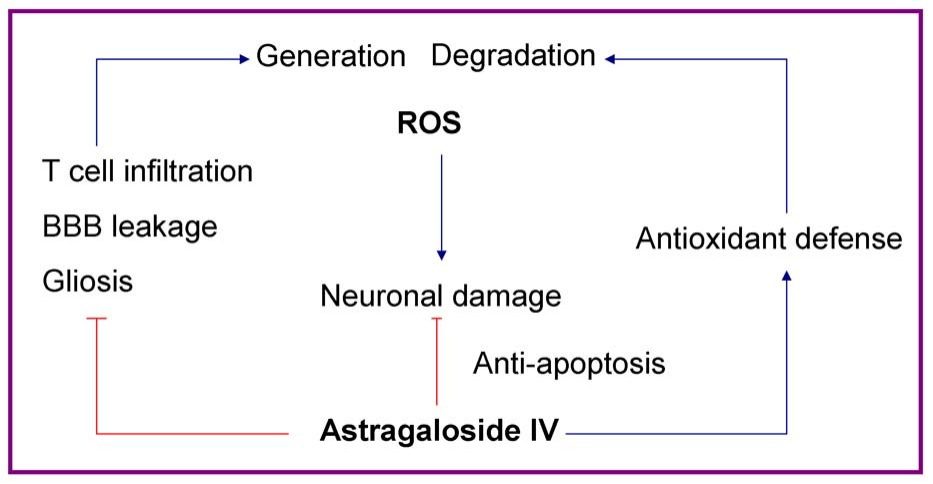
Demonstration of pathways of astragaloside IV to counterbalance oxidative stress in EAE mice. On one hand, ASI enhanced antioxidative defense system and thus elevated ROS degradation capability of CNS. On the other hand, ASI inhibited T-cell infiltration, BBB leakage and gliosis in CNS, thus, reduced ROS generation. ASI regulated ROS generation and degradation and thus decreased its damage to neurons. Meanwhile, ASI could also influence the balance of pro- and anti-apoptotic proteins of Bcl-2 family and make the balance move towards anti-apoptotic direction. ASI, astragaloside IV.

In summary (Fig. 10), alleviation of the progression of EAE by ASI was correlated with its features in anti-oxidation and anti-apoptosis. On one hand, ASI prevented ROS generation by inhibition of T cell infiltration into CNS, BBB leakage and neuroinflammation. On the other hand, ASI enhanced anti-oxidative and anti-apoptotic capability of neuronal cells thus reduced ROS caused damage to nervous system.

## Supporting Information

Figure S1 **Effect of astragaloside IV on CD3 expression in spinal cords of EAE mice.** A, western blots of CD3 and β-actin in spinal cords of EAE mice treated with astragaloside IV. B, gray intensity analysis of CD3. All data are presented as mean±standard error of the mean and compared with EAE group. n = 3 for each group. *, p<0.05; **, p<0.01.
(TIF)

## References

[1] Frohman EM, Racke MK, Raine CS (2006) Multiple sclerosis-the plaque and its pathogenesis. N Engl J Med 354: 942–955. 16510748 10.1056/NEJMra052130

[2] Compston A, Coles A (2008) Multiple sclerosis. Lancet 372: 1502–1517. 18970977 10.1016/S0140-6736(08)61620-7

[3] Frigo M, Cogo MG, Fusco ML, Gardinetti M, Frigeni B (2012) Glutamate and multiple sclerosis. Curr Med Chem 19: 1295–1299. 22304707 10.2174/092986712799462559

[4] Cohen JA (2009) Emerging therapies for relapsing multiple sclerosis. Arch Neurol 66(7): 821–828. 19597083 10.1001/archneurol.2009.104

[5] Castro-Borrero W, Graves D, Frohman TC, Flores AB, Hardeman P, et al (2012) Current and emerging therapies in multiple sclerosis: a systematic review. Ther Adv Neurol Dis 5(4): 205–220. 10.1177/1756285612450936 PMC3388530 22783370

[6] Amorini AM, Petzold A, Tavazzi B, Eikeleniboom J, Keir G, et al (2009) Increased of uric acid and purine compounds in biological fluids of multiple sclerosis patients. Clin Biochem 42: 1001–1006. 19341721 10.1016/j.clinbiochem.2009.03.020

[7] Acar A, Cevik MU, Evliyaoglu S, Uzar E, Tamam Y, et al (2012) Evaluation of serum oxidant/antioxidant balance in multiple sclerosis. Acta Neurol Belg 112: 275–280. 22450709 10.1007/s13760-012-0059-4

[8] Tasset I, Agüera E, Sánchez-López F, Feijóo M, Giraldo AI, et al (2012) Peripheral oxidative stress in relapsing-remitting multiple sclerosis. Clin Biochem 45: 440–444. 22330938 10.1016/j.clinbiochem.2012.01.023

[9] Oliveira SR, Kallaur AP, Simão ANC, Morimoto HK, Lopes J, et al (2012) Oxidative stress in multiple sclerosis patients in clinical remission: association with the expanded disability status scale. J Neurol Sci. http://dx.doi.org/10.1016/j.jns.2012.07.045 10.1016/j.jns.2012.07.045 22883481

[10] Floris S, Blezer ELA, Schreibelt G, Döpp E, van der Pol SMA, et al (2004) Blood-brain barrier permeability and monocyte infiltration in experimental allergic encephalomyelitis: a quantitative MRI study. Brain 127: 616–627. 14691063 10.1093/brain/awh068

[11] Schreibelt G, Musters RJP, Reijerkerk A, de Groot LR, van der Pol SMA, et al (2006) Lipoic acid affects cellular migration into the central nervous system and stabilizes blood-brain barrier integrity. J Immunol 177: 2630–2637. 16888025 10.4049/jimmunol.177.4.2630

[12] van Horssen J, Witte ME, Schreibelt G, de Vries HE (2011) Radical changes in multiple sclerosis pathogenesis. Biochim Biophys Acta 1812: 141–150. 20600869 10.1016/j.bbadis.2010.06.011

[13] Witherick J, Wilkins A, Scolding N, Kemp K (2011) Mechanisms of oxidative damage in multiple sclerosis and a cell therapy approach to treatment. Autoimmune Dis 164608. 10.4061/2011/164608 PMC3010615 21197107

[14] Dringen R (2005) Oxidative and antioxidative potential of brain microglial cells. Antioxid Redox Signal 7: 1223–1233. 16115027 10.1089/ars.2005.7.1223

[15] Gilgun-Sherki Y, Melamed E, Offen D (2004) The role of oxidative stress in the pathogenesis of multiple sclerosis: the need for effective antioxidant therapy. J Neurol 251: 261–268. 15015004 10.1007/s00415-004-0348-9

[16] Ko JK, Lam FY, Cheung AP (2005) Amelioration of experimental colitis by *Astragalus membranaceus* through anti-oxidation and inhibition of adhesion molecule synthesis. World J Gastroenterol 11: 5787–5794. 16270386 10.3748/wjg.v11.i37.5787 PMC4479677

[17] Zhang WJ, Hufnagl P, Binder BR, Wojta J (2003) Antiinflammatory activity of astragaloside IV is mediated by inhibition of NF-kappaB activation and adhesion molecule expression. Thromb Haemost 90: 904–914. 14597987 10.1160/TH03-03-0136

[18] Luo Y, Qin Z, Hong Z, Zhang X, Ding D, et al (2004) Astragaloside IV protects against ischemic brain injury in a murine model of transient focal ischemia. Neurosci Lett 363: 218–223. 15182947 10.1016/j.neulet.2004.03.036

[19] Zhang WD, Zhang C, Wang XH, Gao PJ, Zhu DL, et al (2006) Astragaloside IV dilates aortic vessels from normal and spontaneously hypertensive rats through endothelium-dependent and endothelium-independent ways. Planta Med 72: 621–626. 16732512 10.1055/s-2006-931572

[20] Jiang QL, Jiang JW, Lu XX (2003) Effects of astragaloside IV on glucagon-like peptide-1. Chin J Gerontol 23: 52–53.

[21] Cao Q, Li ZP (2002) Effects of astragaloside IV on myocardial calcium transport and cardiac function in ischemic rats. Acta Pharmacol Sin 23: 898–904. 12370095

[22] Qiu LH, Xie XJ, Zhang BQ (2010) Astragaloside IV improves homocysteine induced acute phase endothelial dysfunction via antioxidation. Biol Pharm Bull 33(4): 641–646. 20410599 10.1248/bpb.33.641

[23] Wang S, Qiu J, Bai Q, Li J, He J, et al (2011) A study on protection of astragaloside IV about oxidative stress on PC12 cells induced by H_2_O_2_ . Chin Pharmacol Bull 27(11): 1603–1609.

[24] Wang Y, Peng Y, Zhang Q, Wu Y, Song J, et al (2011) Effect of ERK1/2 signaling pathway on astragaloside IV protects H9c2 cells against H_2_O_2_-induced oxidative injury. Chin J App Physiol 27(3): 363–367. 22097737

[25] Cheng CY, Yao CH, Liu BS, Liu CJ, Chen GW, et al (2006) The role of astragaloside in regeneration of the peripheral nerve system. J Biomed Mater Res A 76: 463–469. 16315188 10.1002/jbm.a.30249

[26] Wu X, Pan W, He Y, Hsuchou H, Kastin AJ (2010) Cerebral interleukin-15 shows upregulation and beneficial effects in experimental autoimmune encephalomyelitis. J Neuroimmunol 223: 65–72. 20430449 10.1016/j.jneuroim.2010.04.001 PMC2883030

[27] Peiris M, Monteith GR, Roberts-Thomson SJ, Cabot PJ (2007) A model of experimental autoimmune encephalomyelitis (EAE) in C57BL/6 mice for the characterisation of intervention therapies. J Neurosci Meth 163(2): 245–254. 10.1016/j.jneumeth.2007.03.013 17477973

[28] Schreibelt G, van Horssen J, Haseloff RF, Reijerkerk A, van der Pol SM, et al (2008) Protective effects of peroxiredoxin-1 at the injured blood-brain barrier. Free Radic Biol Med 45(3): 256–264. 18452719 10.1016/j.freeradbiomed.2008.03.024

[29] Uzar E, Koyuncuoglu HR, Uz E, Yilmaz HR, Kutluhan S, et al (2006) The activities of antioxidant enzymes and the level of malondialdehyde in cerebellum of rats subjected to the level of malondialdehyde in cerebellum of rats subjected to methotrexate: protective effect of caffeic acid phenethyl ester. Mol Cell Biochem 291: 63–68. 16718360 10.1007/s11010-006-9196-5

[30] Koch M, Ramsaransing GSM, Arutjunyan AV, Stepanov M, Teelken A, et al (2006) Oxidative stress in serum and peripheral blood leukocytes in patients with different disease courses of multiple sclerosis. J Neurol 253: 483–487. 16283096 10.1007/s00415-005-0037-3

[31] Kuhlmann CRW, Tamaki R, Gamerdinger M, Lessmann V, Behl C, et al (2007) Inhibition of the myosin light chain kinase prevents hypoxia-induced blood-brain barrier disruption. J Neurochem 102: 501–507. 17419808 10.1111/j.1471-4159.2007.04506.x

[32] Lagrange P, Romero IA, Minn A, Revest PA (1999) Transendothelial permeability changes induced by free radicals in an in vitro model of the blood-brain barrier. Free Radic Biol Med 27: 667–672. 10490287 10.1016/s0891-5849(99)00112-4

[33] van Horssen J, Schreibelt G, Drexhage J, Hazes T, Dijkstra CD, et al (2008) Severe oxidative damage in multiple sclerosis lesions coincides with enhanced antioxidant enzyme expression. Free Radic Biol Med 45(12): 1729–1737. 18930811 10.1016/j.freeradbiomed.2008.09.023

[34] Kuchroo VK, Anderson AC, Waldner H, Munder M, Bettelli E, et al (2002) T cell response in experimental autoimmune encephalomyelitis (EAE): role of self and cross-reactive antigens in shaping, tuning, and regulating the autopathogenic T cell repertoire. Annu Rev Immunol 20: 101–123. 11861599 10.1146/annurev.immunol.20.081701.141316

[35] Almolda B, Costa M, Montoya M, González B, Castellano B (2011) Increase in Th17 and T-reg lymphocytes and decrease of IL22 correlate with the recovery phase of acute EAE in rat. PLoS One 6(11): e27473 doi: 10.1371/journal.pone.0027473 22110656 10.1371/journal.pone.0027473 PMC3217052

[36] Szabo SJ, Kim ST, Costa GL, Zhang X, Fathman CG, et al (2000) A novel transcription factor, T-bet, directs Th1 lineage commitment. Cell 100: 655–669. 10761931 10.1016/s0092-8674(00)80702-3

[37] Miller SA, Weinmann AS (2009) Common themes emerge in the transcriptional control of T helper and developmental cell fate decisions regulated by the T-box, GATA and ROR families. Immunology 126: 306–315. 19302139 10.1111/j.1365-2567.2008.03040.x PMC2669811

[38] Huang LF, Yao YM, Li JF, Zhang SW, Li WX, et al (2012) The effect of Astragaloside IV on immune function of regulatory T cell mediated by high mobility group box 1 protein in vitro. Fitoterapia 83(8): 1514–1522. 22981502 10.1016/j.fitote.2012.08.019

[39] Zargari M, Allameh A, Sanati MH, Tiraihi T, Lavasani S, et al (2007) Relationship between the clinical scoring and demyelination in central nervous system with total antioxidant capacity of plasma during experimental autoimmune encephalomyelitis development in mice. Neurosci Lett 412: 24–28. 17157437 10.1016/j.neulet.2006.08.033

[40] Cory S, Adams JM (2002) The Bcl-2 family: regulators of the cellular life-or-death switch. Nat Rev Cancer 2: 647–656. 12209154 10.1038/nrc883

[41] Tanaka K, Asanuma M, Ogawa N (2004) Molecular basis of anti-apoptotic effect of immunophilin ligands on hydrogen peroxide-induced apoptosis in human glioma cells. Neurochem Res 29: 1529–1536. 15260130 10.1023/b:nere.0000029565.92587.25

[42] Levy OA, Malagelada C, Greene LA (2009) Cell death pathways in Parkinson’s disease: proximal triggers, distal effectors, and final steps. Apoptosis. 14: 478–500. 10.1007/s10495-008-0309-3 PMC2754154 19165601

[43] Dimmeler S, Breitschopf K, Haendeler J, Zeiher AM (1999) Dephosphorylation targets Bcl-2 for ubiquitin-dependent degradation: a link between the apoptosome and the proteasome pathway. J Exp Med 189(11): 1815–1822. 10359585 10.1084/jem.189.11.1815 PMC2193081

[44] Shaw PH (1996) The role of p53 in cell cycle regulation. Pathol Res Pract 192: 669–675. 8880867 10.1016/S0344-0338(96)80088-4

[45] Meβmer UK, Ankarcrona M, Nicotera P, Brüne B (1994) p53 expression in nitric oxide-induced apoptosis. FEBS Lett 355: 23–26. 7525358 10.1016/0014-5793(94)01161-3

[46] Forrester K, Ambs S, Lupold SE, Kapust RB, Spillare EA, et al (1996) Nitric oxide-induced p53 accumulation and regulation of inducible nitric oxide synthase expression by wild-type p53. Proc Natl Acad Sci 93: 2442–2447. 8637893 10.1073/pnas.93.6.2442 PMC39816

[47] Miyashita T, Krajewski S, Krajewska M, Wang HG, Lin HK, et al (1994) Tumor suppressor p53 is a regulator of bcl-2 and bax gene expression in vitro and in vivo. Oncogene 9(6): 1799–1805. 8183579

[48] Xiang H, Kinoshita Y, Knudson CM, Korsmeyer SJ, Schwartzkroin PA, et al (1998) Bax involvement in p53-mediated neuronal cell death. J Neurosci 18(4): 1363–1373. 9454845 10.1523/JNEUROSCI.18-04-01363.1998 PMC6792710

[49] Lee VM, Goedert M, Trojanowski JQ (2001) Neurodegenerative tauopathies. Annu Rev Neurosci 24: 1121–1159. 11520930 10.1146/annurev.neuro.24.1.1121

[50] Ebneth A, Godemann R, Stamer K, Illenberger S, Trinczek B, et al (1998) Overexpression of tau protein inhibits kinesin-dependent trafficking of vesicles, mitochondria, and endoplasmic reticulum: implications for Alzheimer’s disease. J Cell Biol 143(3): 777–794. 9813097 10.1083/jcb.143.3.777 PMC2148132

[51] Schneider A, Araújo GW, Trajkovic K, Herrmann MM, Merkler D, et al.. (2004) Hyperphosphorylation and aggregation of tau in experimental autoimmune encephalomyelitis. J Biol Chem 55833–55839. 10.1074/jbc.M409954200 15494405

[52] Anderson JM, Hampton DW, Patani R, Pryce G, Crowther RA, et al (2008) Abnormally phosphorylated tau is associated with neuronal and axonal loss in experimental autoimmune encephalomyelitis and multiple sclerosis. Brain 131: 1736–1748. 18567922 10.1093/brain/awn119

[53] Chen LQ, Wei JS, Lei ZN, Zhang LM, Liu Y, et al (2005) Induction of Bcl-2 and Bax was related to hyperphosphorylation of tau and neuronal death induced by okadaic acid in rat brain. Anat Rec A Discov Mol Cell Evol Biol 287(2): 1236–45. 16265626 10.1002/ar.a.20241

[54] Kreutzberg GW (1996) Microglia: a sensor for pathological events in the CNS. Trends Neurosci 19: 312–318. 8843599 10.1016/0166-2236(96)10049-7

[55] Glass CK, Saijo K, Winner B, Marchetto MC, Gage FH (2010) Mechanisms underlying inflammation in neurodegeneration. Cell 140: 918–934. 20303880 10.1016/j.cell.2010.02.016 PMC2873093

[56] Spencer JPE, Vafeiadou K, Williams RJ, Vauzour D (2012) Neuroinflammation: modulation by flavonoids and mechanisms of action. Mol Aspects Med 33: 83–97. 22107709 10.1016/j.mam.2011.10.016

[57] Zhang Z, Wu L, Wang J, Yang J, Zhang J, et al (2012) Astragaloside IV prevents MPP+-induced SH-SY5Y cell death via the inhibition of Bax-mediated pathways and ROS production. Mol Cell Biochem 364: 209–216. 22278385 10.1007/s11010-011-1219-1

[58] Chan W, Durairajan SSK, Lu J, Wang Y, Xie L, et al (2006) Neuroprotective effects of astragaloside IV in 6-hydroxydopamine-treated primary nigral cell culture. Neurochem Int 55: 414–422. 10.1016/j.neuint.2009.04.012 19409437

[59] Zhu SQ, Qi L, Rui YF, Li RX, He XP, et al (2008) Astragaloside IV inhibits spontaneous synaptic transmission and synchronized Ca^2+^ oscillations on hippocampal neurons. Acta Pharmacol Sin 29: 57–64. 18158866 10.1111/j.1745-7254.2008.00712.x

