# Supplementary figures and images for: Astragaloside IV Attenuates Experimental Autoimmune Encephalomyelitis of Mice by Counteracting Oxidative Stress at Multiple Levels
Source: PLoS One. 2013 Oct 4;8(10):e76495. doi: 10.1371/journal.pone.0076495 (PMC3790693; doi:10.1371/journal.pone.0076495)

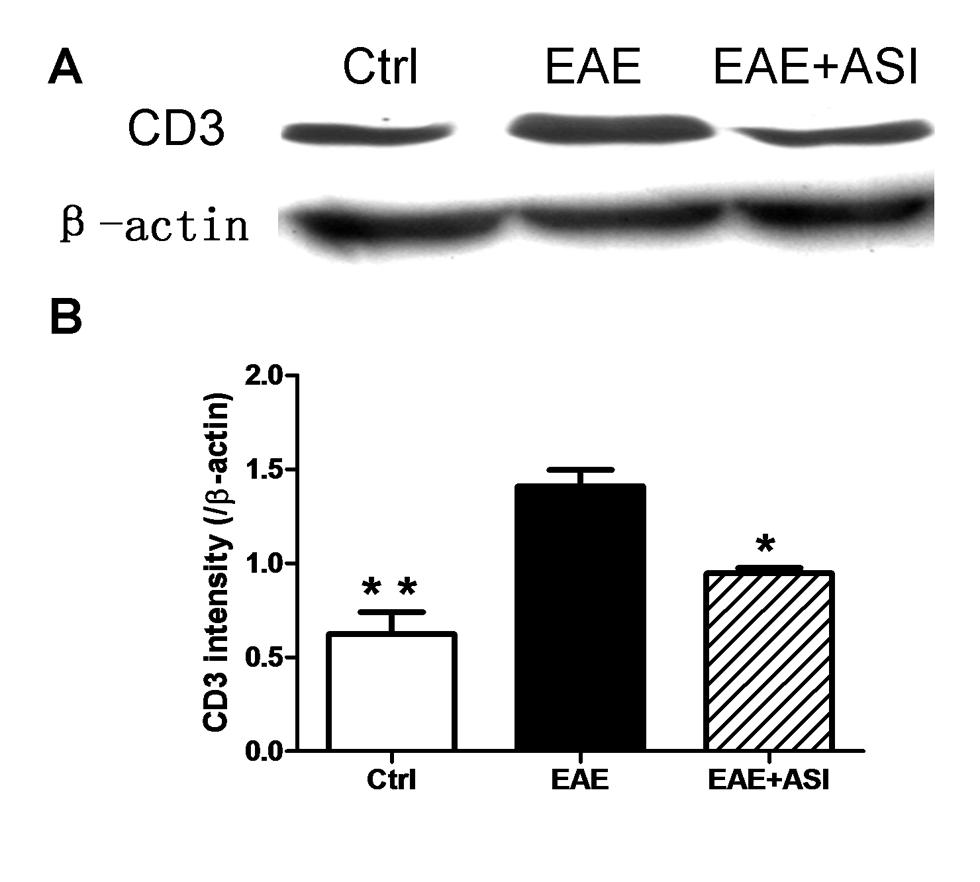

Supplement: Figure S1 — Effect of astragaloside IV on CD3 expression in spinal cords of EAE mice. A, western blots of CD3 and β-actin in spinal cords of EAE mice treated with astragaloside IV. B, gray intensity analysis of CD3. All data are presented as mean±standard error of the mean and compared with EAE group. n = 3 for each group. *, p<0.05; **, p<0.01. (TIF) [file pone.0076495.s001.tif]
